# Synthesis and crystal structure of HDAC6 selective inhibitor of *N*-hy­droxy-4-{2-[(2-hy­droxy­eth­yl)(phen­yl)amino]-2-oxoeth­yl}benzamide monohydrate (HPOB·H_2_O)

**DOI:** 10.1107/S2056989025010989

**Published:** 2026-01-06

**Authors:** Zola Cervantes, Lauren Bradford, Andressa Antonini Bertolazzo, Adaickapillai Mahendran, S. Chantal E. Stieber

**Affiliations:** a Department of Chemistry & Biochemistry California State Polytechnic University, Pomona 3801 W Temple Ave Pomona CA 91768 USA; Illinois State University, USA

**Keywords:** crystal structure, HPOB, HDAC6 inhibitor, inhibitors, cancer

## Abstract

The synthesis and crystal structure of the title compound *N*-hy­droxy-4-{2-[(2-hy­droxy­eth­yl)(phen­yl)amino]-2-oxoeth­yl}benzamide monohydrate (**HPOB**·**H_2_O**) is reported. The water mol­ecule is positionally disordered at 106 K. The complex crystallizes with monoclinic *P*2_1_/*n* symmetry, and the core of the mol­ecule is relatively planar with the two aryl substituents rotated out of the plane. This structure highlights how **HPOB**·**H_2_O** has a hydroxamate moiety that adopts a *Z* conformation.

## Abstract 2

The crystal structure of the title compound *N*-hy­droxy-4-{2-[(2-hy­droxy­eth­yl)(phen­yl)amino]-2-oxoeth­yl}benzamide monohydrate (**HPOB**·**H_2_O**) is reported.

## 1. Chemical context

The regulation of gene expression is significantly influenced by histone acetyl­ation, a reversible modification governed by two opposing classes of enzymes: histone acetyl­transferases (HATs) and histone de­acetyl­ases (HDACs). HATs facilitate gene transcription by adding acetyl groups to histone proteins, thereby loosening chromatin structure and enhancing DNA accessibility. Conversely, HDACs remove these acetyl groups, resulting in a more compact chromatin arrangement that restricts transcription. Abnormal patterns of histone acetyl­ation, particularly due to altered HDAC activity, are commonly associated with the development and progression of cancer, making HDACs attractive targets for therapeutic inter­vention (Kim *et al.*, 2020 ▸; Chen *et al.*, 2015 ▸).

Among the eleven zinc-dependent HDAC isoforms found in humans, HDAC6 is particularly notable for its distinct structural features and its involvement in diverse cellular processes such as protein turnover, cytoskeletal dynamics, and response to cellular stress (Kwon *et al.*, 2012 ▸). Designing selective inhibitors for specific HDAC isoforms is critical for elucidating their individual biological roles and minimizing adverse effects linked to non-selective inhibition (Rastelli & Micelli, 2015 ▸). To date, several HDAC6 inhibitors have been developed with selectivities ranging from tenfold inhibition to more than a thousandfold inhibition relative to HDAC1. The HDAC6 inhibitor *N*-Hy­droxy-4-{2-[(2-hy­droxy­eth­yl)(phen­yl)amino]-2-oxoeth­yl}benzamide (**HPOB**) has 52-fold selectivity for inhibition over HDAC1 (Lee *et al.*, 2013 ▸).

The co-crystal structure of HPOB complexed with HDAC6 (*Danio rerio*) enzyme was reported (Hai & Christianson, 2016 ▸), however a standalone crystal structure of HPOB is not yet reported. The co-crystal structure of HPOB-HDAC6 complex has an unusual monodentate Zn^2+^ coordination from HPOB. It is also reported that the HDAC inhibitor tricostatin A complexes with the HDAC6 Zn^2+^ binding pocket in two conformers. A major conformer (70%) with a canonical bidentate hydroxamate- Zn^2+^ coordination geometry and a minor conformer (30%) with monodentate hydroxamate-Zn^2+^ coordination geometry (Porter, *et al.*, 2017 ▸). The ^1^H NMR spectra of a pyrimidine-based hydroxamic acid in DMSO-*d*_6_ have shown two sets of proton signals from NH and OH groups representing *E* and *Z* forms (Jakubkiene *et al.*, 2022 ▸). It is also reported elsewhere that the ratio of Z to E isomer decreases in the order of DMSO-*d*_6_, < CDCI_3_, < C_6_D_6_ (Brown *et al.*, 1991 ▸, 1996 ▸; Sow *et al.*, 2023*a* ▸,*b* ▸). This suggests that the *Z* isomer is preferentially stabilized in DMSO-*d*_6_ (potentially through water inter­actions), while the *E* isomer becomes more stable in non-polar hydro­carbon solvents.

In this work, we report the synthesis and crystal structure of **HPOB**·**H_2_O**, a hydroxamic acid-based compound known for its selectivity toward HDAC6. Our findings disclose the single crystal X-ray structure of **HPOB**·**H_2_O**, which adopts a *Z* conformation. These structural insights provide valuable information about how **HPOB** may inter­act with metal ions at the active site of HDAC6 and other HDAC enzyme, contributing to a better understanding of its binding mode and offering guidance for the development of related inhibitors.

## 2. Structural commentary

**Figure 1:**
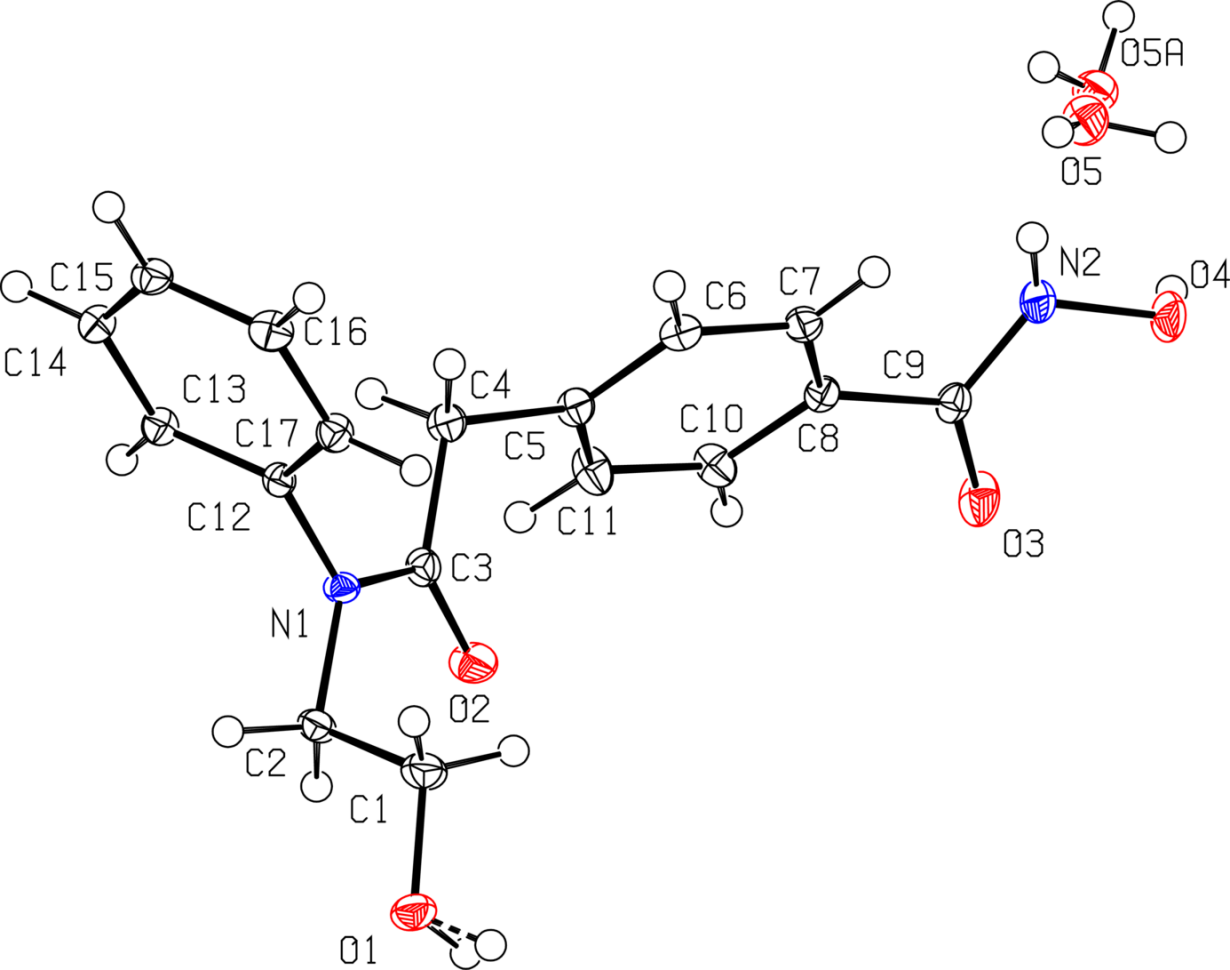
View of HPOB·H_2_O with 50% probability ellipsoids, showing the H_2_O disorder.

**HPOB·H_2_O** crystallizes with one mol­ecule of HPOB and one mol­ecule of water within the asymmetric unit, as depicted in Fig. 1 ▸. The core chain of the mol­ecule has bond distances consistent with single bonds for C1—C2 at 1.520 (3) Å, C2—N1at 1.470 (3) Å, N1—C3 at 1.356 (3) Å, C3—C4 at 1.525 (3) Å, and C4—C5 at 1.509 (3) Å. The bonds to oxygen atoms have bond lengths consistent with terminal OH groups for O1—C1 at 1.434 (3) Å and N2—O4 at 1.397 (2) Å, and a bond distance consistent with a ketone for C9—O3 at 1.239 (3) Å. The core of the HPOB mol­ecule is relatively planar between C2, N1, C3, C4, and C5 with the two aryl rings rotated out of the plane. The aryl rotation is reflected by the torsion angles C3—C4—C5—C6 of −113.8 (2)° and C3—N1—C12—C17 at −98.3 (2)°. The hydroxamate group adopts a *Z* conformation, similar to that observed in the co-crystal structure of HPOB bound to the HDAC6 enzyme reported by Hai & Christianson (2016 ▸). In this conformation, the hydroxamate hydroxyl group coordinates with the enzyme’s Zn^2+^ cofactor through Zn^2+^-bound water mol­ecule, which remains undisplaced.

## 3. Supra­molecular features

**Figure 2:**
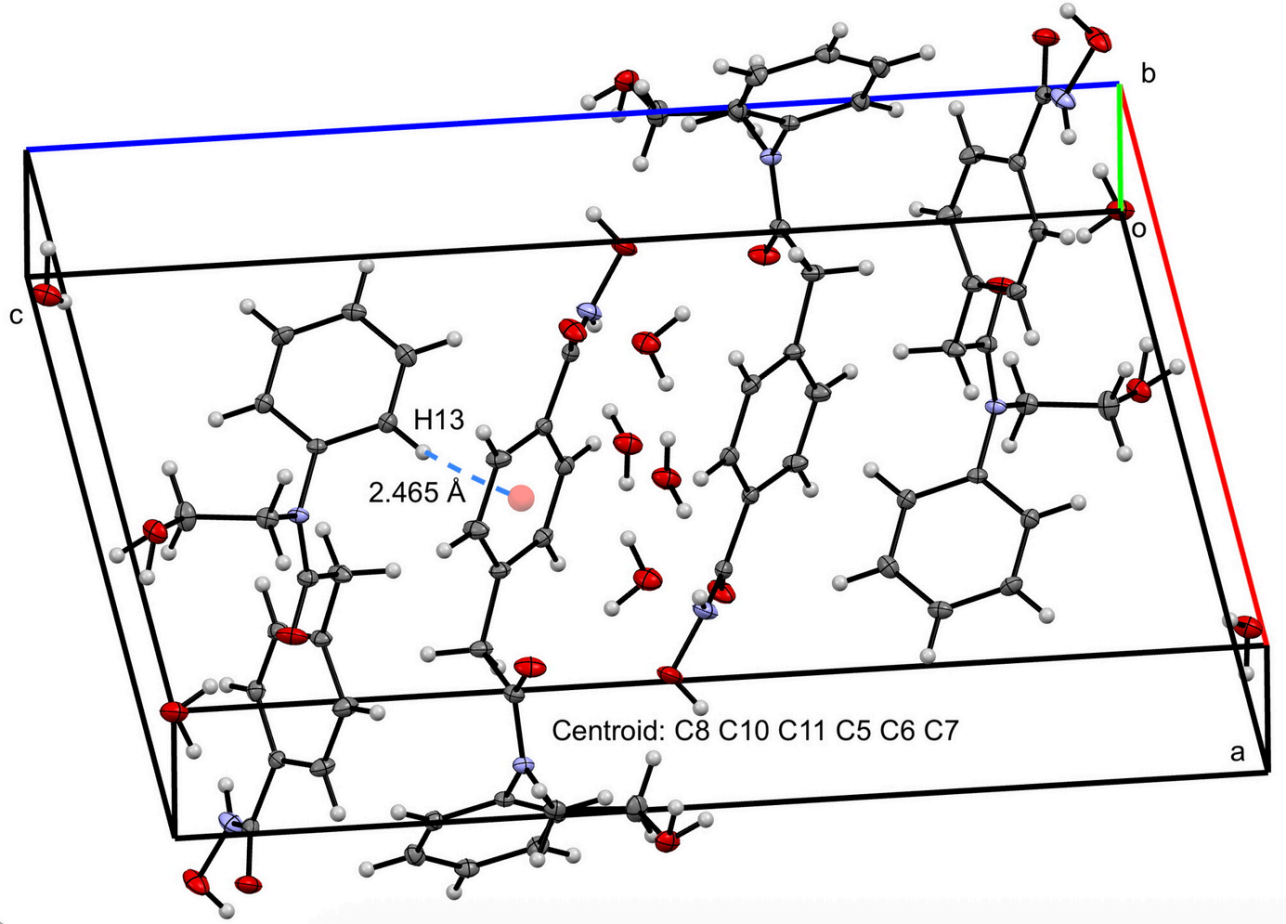
View of four mol­ecules of HPOB and H_2_O in the unit cell with 50% probability ellipsoids, highlighting the inter­molecular π inter­action. Distances between H atoms are listed without standard deviations because the H atoms were positionally fixed.

**Figure 3:**
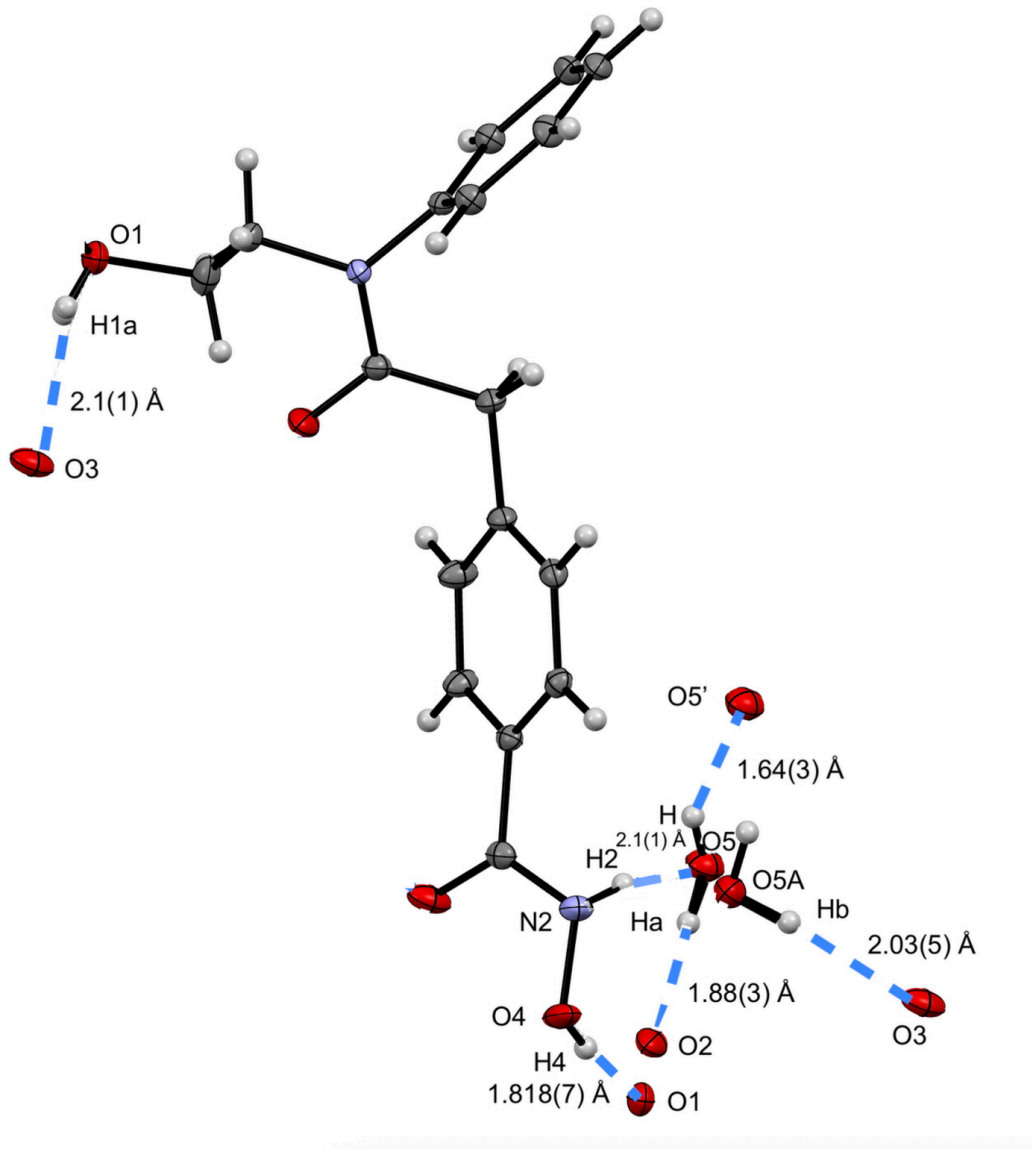
View of one mol­ecule of HPOB·H_2_O highlighting hydrogen-bonding inter­actions from neighboring mol­ecules.

Four mol­ecules of HPOB and water are in the unit cell as depicted in Fig. 2 ▸ with primary stabilization from hydrogen bonding (Table 1 ▸), and perpendicular π stacking. The perpendicular π stacking is apparent from measuring a distance from the centroid between C5–C6–C7–C8–C10–C11 and H13 of 2.465 Å. Hydrogen-bonding distances and angles are reported in Table 1 ▸ and are highlighted in Fig. 3 ▸. The two hydroxide moieties in the mol­ecule have hydrogen bonds to a neighboring mol­ecule of HPOB with a O1—H1*a*⋯O3 hydrogen-bond distance of 2.1 (1) Å to a second hydroxide, and a O4—H4⋯O1 hydrogen-bond distance of 1.818 (7) Å to the carbonyl. The water mol­ecule in the structure also is involved in hydrogen bonding to the amino group of HPOB with an N2—H2⋯O5 hydrogen-bond distance of 2.05 (4) Å, to the carbonyls of HPOB with a O5—H*a*⋯O2 hydrogen-bond distance of 1.88 (3) Å and a O5*A*—H*b*⋯O3 hydrogen-bond distance of 2.03 (5) Å, and with another water mol­ecule with a O5—H⋯O5′ hydrogen-bond distance of 1.64 (3) Å.

## 4. Database survey

A survey of Cambridge Structural Database (WebCSD accessed July 29, 2025; Groom *et al.*, 2016 ▸) and Scifinder (SciFinder, 2025) yielded no exact matches for a standalone structure for HPOB. However, the crystal structure of the HPOB complexed with the HDAC6 enzyme has been reported (Hai & Christianson, 2016 ▸), in which the hydroxamate group is in the Z conformation and the C=O group forms a hydrogen bond with a Zn^2+^-bound water mol­ecule. Two of the precursors to HPOB are also reported in the Cambridge Structural Database including Compound 1 (Saeed *et al.*, 2008 ▸) and Compound 2 (Yathirajan *et al.*, 2007 ▸).

## 5. Synthesis and crystallization

**Figure 4:**
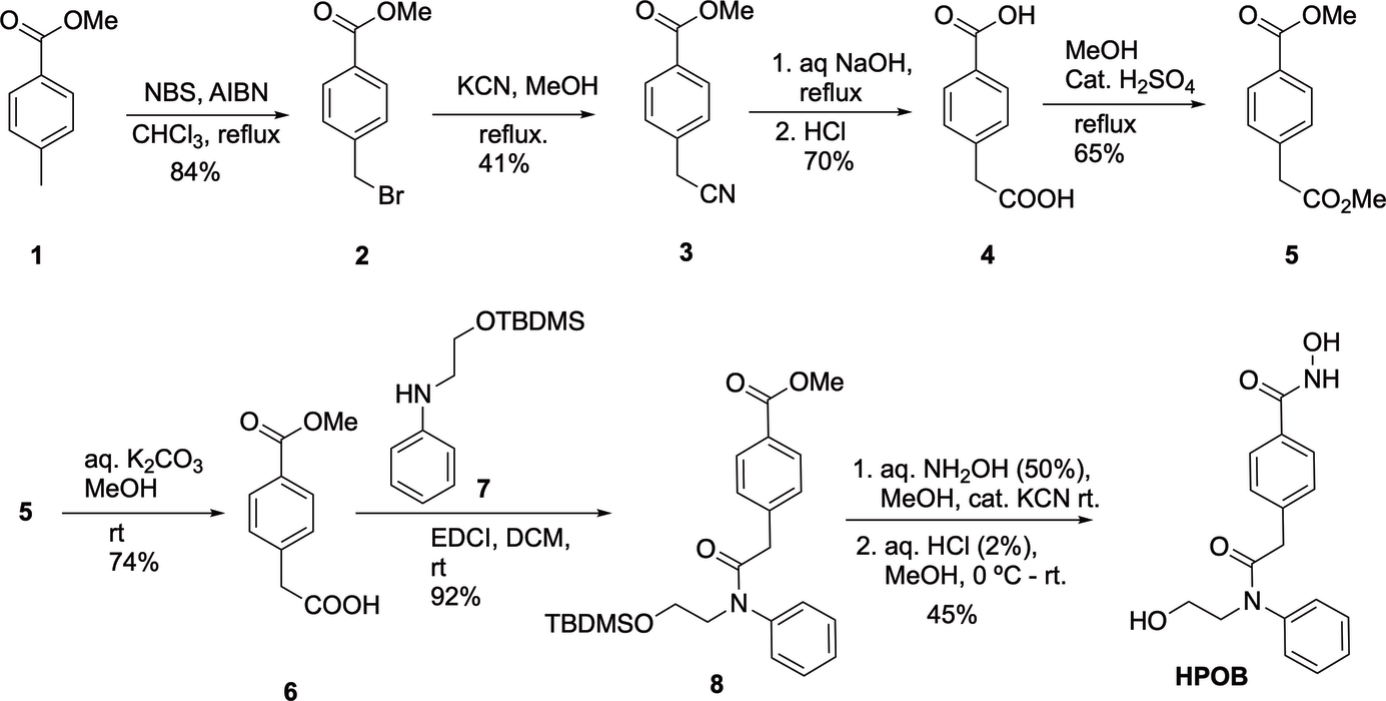
Synthetic scheme for the synthesis of HPOB.

**General** c**onsiderations.** All reagents were purchased from commercial suppliers and used without further purification unless otherwise noted. ^1^H, and ^13^C NMR spectra were recorded on a Varian 400 MHz instrument operating at 399.7770024 MHz. Chemical shifts are reported in ppm relative to SiMe_4_. Spectra were processed using MestReNova and original files and NMR data can be accessed through Zenodo (Cervantes *et al.*, 2025 ▸). The synthesis is shown in Fig. 4 ▸.

**HPOB·H_2_O** (*N*-Hy­droxy-4-{2-[(2-hy­droxy­eth­yl)(phen­yl)amino]-2-oxoeth­yl}benzamide) was synthesized from methyl-*p*-toluate (**1**) in seven steps with an overall yield of 4.8%. First, compound **1** was brominated with NBS to produce methyl 4-(bromo­meth­yl)benzoate, compound **2** (Takahashi *et al.*, 2008 ▸). Then, compound **2** was reacted with KCN in methanol to yield the cyano compound **3** (Sakellariou *et al.*, 2003 ▸), which was subsequently hydrolyzed to produce the diacid **4** (Saraswati *et al.*, 2020 ▸). Compound **4** was then subjected to an esterification reaction with methanol to yield diester **5** (Saraswati *et al.*, 2020 ▸) Then diester **5** was selectively hydrolyzed at the aliphatic ester to form monoester compound **6 (**Saraswati *et al.*, 2020 ▸). Then, compound **6** was subjected to amide coupling with TBDMS-protected phenyl amino ethanol (**7**) (Zhao *et al.*, 2021 ▸), in the presence of EDCI to produce amide **8**. Lastly, amide **8** was reacted with 50% aqueous hydroxyl­amine solution in the presence of a catalytic amount of KCN, and then hydrolyzed with HCl to produce the target compound **HPOB. HPOB·H_2_O** was crystallized in 95% ethanol *via* slow evaporation over a period of two days.

**Methyl-4-(bromo­meth­yl)benzoate (2):***N*-Bromo­succin­im­ide (6.6 g, 37.1 mmol, 1.3 eq) and azobisisobutyro­nitrile (433 mg, 2.6 mmol, 0.1 eq) were added to a solution of methyl-*p*-toluate (**1**, 4.3 g, 28.5 mmol, 1 eq) and 260 mL of chloro­form. The reaction mixture was refluxed for 24 h under an argon atmosphere. After cooling the reaction mixture to room temperature the solvent was evaporated and the white solid was dissolved in ethyl acetate (200 mL). The organic layer was washed with brine solution (100 mL) and then water (100 mL), dried with anhydrous Na_2_SO_4_, filtered, concentrated *in vacuo*. The crude product was chromatographed on silica gel (hexa­nes/EtOAc, 9:1) to yield target compound **2**. Yield 5.5 g, (84%); ^1^H NMR (CHCl_3_, 400 MHz): δ 8.02 (*d*, *J* = 8, Hz, 2H), 7.46 (*d*, *J* = 8, Hz, 2H), 4.50 (*s*, 2H), 3.92 (*s*, 3H); ^13^C NMR (CDCl_3_, 100 MHz): δ 166.5, 142.6, 130.1, 129.0, 126.6, 52.2, 32.2.

**Methyl 4-(cyano­meth­yl)benzoate (3):** Potassium Cyanide (1.54 g, 23.4 mmol, 1.1 eq) was dissolved in water (5 mL), and subsequently added to the methyl 4-bromo­methyl benzoate **2** (4.7 g, 20.5 mmol 1 eq) in 40 mL of methanol. The solution was then refluxed for 24 h at 333 K. The resulting solution was concentrated and extracted with diethyl ether (2 × 40 mL). The combined organic layers were washed with 30 mL of H_2_O, dried with anhydrous Na_2_SO_4_, filtered, and concentrated *in vacuo*. The crude product was chromatographed on silica gel (hexa­nes/EtOAc, 7:3) to yield target compound **3**. Yield 1.50g (41%); ^1^H NMR (400 MHz, CDCl3): δ 8.05 (*d*, *J* = 8, Hz, 2H), 7.42 (*d*, *J* = 8, Hz, 2H), 3.93 (*s*, 3H), 3.81 (s, 2H); ^13^C NMR (101 MHz, CDCl_3_) δ 166.4, 134.8, 130.4, 128.0, 117.1, 77.3, 77.0, 76.7, 52.3, 23.7, 23.6.

**4-(carb­oxy­meth­yl)benzoic acid (4):** In a solution of methanol (20 mL) and 6 *M* sodium hydroxide (20 mL), compound **3** (2.6 g, 11.3 mmol) was added to a round-bottom flask equipped with a magnetic stirrer. The solution was subsequently heated under reflux at 363 K for about 24 h. After cooling it to room temperature the solution was concentrated to remove methanol solvent. The aqueous layer was acidified with hydro­chloric acid to a pH of 1–2, and the product was extracted with CH_2_Cl_2_ (35 mL × 2). Combined organic layers was washed with 30 mL H_2_O, dried with anhydrous Na_2_SO_4_, filtered, concentrated *in vacuo*. Yield 1.9 g (70%); ^1^H NMR (400 MHz, acetone): δ 7.99 (*d*, *J* = 8, Hz, 2H), 7.47 (*d*, *J* = 8, Hz, 2H), 3.74 (*s*, 2H).

**Methyl 4-(2-meth­oxy-2-oxoeth­yl)benzoate (5):** Compound **4** (1.4 g, 7.8 mmol) was heated at reflux with conc. H_2_SO_4_ (3 mL) as a catalyst, in a methanol (30 mL) solvent at 363 K for 18 h. After cooling to room temperature, the solvent was evaporated *in vacuo*. Ethyl acetate (25 mL) was added to the reaction mixture and then the organic layer was washed with NaHCO_3_ (25 mL). The organic layer was then dried with Na_2_SO_4_, filtered and concentrated *in vacuo*. Yield: 1.06g (65%); ^1^H NMR (400 MHz, CDCl3): δ 8.99 (*d*, *J* = 8, Hz, 2H), 7.33 (*d*, *J* = 8, Hz, 2H), 3.89 (*s*, 3H), 3.68 (*s*, 3H), 3.55 (*s*, 2H); ^13^C NMR (100 MHz, CDCl_3_) δ 170.1, 165.4, 138.8, 128.4, 128.0, 127.8, 52.3, 52.1, 42.3.

**2-[4-(meth­oxy­carbon­yl)phen­yl]acetic acid (6):** K_2_CO_3_ (1.0 g, 7.24 mmol) was added to a solution of compound **5** (0.9 g, 4.28 mmol) and 1:1 mixture of H_2_O/Methanol (31 mL). The reaction mixture was stirred overnight at room temperature. Following this, it was concentrated *in vacuo* and subsequently diluted with H_2_O (25 mL). The mixture was washed with CH_2_Cl_2_ (25 mL × 2), acidified with HCl to pH 3, and then extracted with ethyl acetate (25 mL × 2). The combined organic layer was then dried with Na_2_SO_4_, filtered and concentrated *in vacuo*. Yield: 616 mg (74%); ^1^H NMR (CHCl_3_, 400 MHz): δ 8.03 (*d*, *J* = 8 Hz, 2H), 7.35 (*d*, *J* = 8 Hz, 2H), 3.91 (*s*, 3H), 3.71 (*s*, 2H); ^13^C NMR (CHCl_3_, 100 MHz): δ 176.4, 166.8, 138.3, 129.9, 129.5, 129.3, 52.1, 40.8.

***N*****-{2-[(*****tert*****-butyl­dimethyl­sil­yl)­oxy]eth­yl}aniline (7).** TBDMS-Cl (1.28 g, 8.02 mmol) and imidazole (1.45 g, 21.86 mmol) was added to a solution containing 2-(phenyl­amino) ethanol, (1.00 g, 7.29 mmol) in CH_2_Cl_2_ (20 mL). The reaction mixture was stirred at room temperature in an argon atmosphere for 3 h. Then the reaction was quenched with sat. NH_4_Cl. (20 mL) and CH_2_Cl_2_ (20 mL) and then washed with brine (20 mL). The organic layer was dried (anhydrous sodium sulfate) and concentrated *in vacuo* to yield target compound **7**. Yield 1.82 g, 99%. ^1^H NMR (CHCl_3_, 400 MHz): δ 7.22 (*dd*, *J* = 8.8, 7.4 Hz, 2H), 6.76 (*t*, *J* = 7.4 Hz, 1H), 6.68 (*d*, *J* = 8.8, 2H), 4.09 (*br s*, 1H), 3.86 (*t*, *J* = 5.2 Hz, 2H), 3.26 (*t*, *J* = 5.2 Hz, 2H), 0.95 (*s*, 9H), 0.11 (*s*, 6H); ^13^C NMR (CHCl_3_, 100 MHz): δ 148.4, 129.2, 117.5, 113.2, 61.6, 46.0, 25.9, 18.3, −5.3.

**Methyl 4-(2-[{2-[(*****tert*****-butyl­dimethyl­sil­yl)­oxy]eth­yl}[(phen­yl)αmino]-2-oxoeth­yl} benzoate (8).** EDCI (114.5 mg, 0.60 mmol) was added to a solution containing *N*-{2-[(*tert*-butyl­dimethyl­sil­yl)­oxy]eth­yl}aniline, (100 mg, 0.398 mmol) and 2-(4-(meth­oxy­carbon­yl)phen­yl)acetic acid, **7**, (116 mg, 0.597 mmol) in CH_2_Cl_2_ (3 mL). The reaction mixture was stirred overnight at room temperature in argon atmosphere. After completion of the reaction, the reaction mixture was diluted with mixed solvent (CHCl_3_:*i*-PrOH = 4:1, 10 mL) and washed with sat. NH_4_Cl. The organic layer was dried with Na_2_SO_4_, filtered, and concentrated *in vacuo.* The crude product was chromatographed on silica gel (hexa­nes/EtOAc, 7:1) to yield target compound **8**. Yield 306 mg (90%); ^1^H NMR (CHCl_3_, 400 MHz): δ 7.91 (*d*, *J* = 8.2, Hz, 2H), 7.38 (*m*, 3H), 7.15 (*m*, 4H), 3.91 (*s*, 3H), 3.80 (*m*, 4H), 3.50 (*s*, 2H), 0.85 (*s*, 9H), 0.02 (*s*, 6H); ^13^C NMR (CHCl_3_, 100 MHz): δ 170.2, 167.0, 142.9, 140.8, 129.6, 129.5, 129.1, 128.6, 128.5, 128.0, 60.1, 52.1, 52.0, 41.4, 25.8, 18.2, −5.4.

***N*****-Hy­droxy-4-{2-[(2-hy­droxy­eth­yl)(phen­yl)amino]-2-oxo­eth­yl}benzamide monohydrate (HPOB).** Hydroxyl­amine (0.5 mL, 50% water solution) was added to a solution containing compound **8**, (60 mg, 0.140 mmol) in THF/MeOH (1:1, 1 mL). The reaction mixture was treated with a cat. amount of KCN (∼0.5 mg) and stirred at room temperature in argon atmosphere for 16 h. Then solution was acidified by NH_4_Cl/HCl solution to pH 4. The mixture was diluted with mixed solvent (CHCl_3_:i-PrOH = 4:1, 10 mL) and washed with sat. NH_4_Cl. The organic layer was dried with Na_2_SO_4_, filtered, and concentrated *in vacuo.* Resulted mixture was dissolved in 2% HCl in EtOH (5 mL) and stirred for 3 h. Then the reaction mixture was concentrated *in vacuo.* The crude product was chromatographed on silica gel (CH_2_Cl_2_/MeOH, 10:1) to yield target compound **HPOB**. Yield 20 mg, 45%. ^1^H NMR (CD_3_OD, 400 MHz): δ 7.61 (*d*, *J* = 8.4 Hz, 2H), 7.43 (*m*, 3H), 7.27 (*d*, *J* = 8.0 Hz, 2H), 7.11(*d*, *J* = 8.0 Hz, 2H), 3.82 (*t*, *J* = 6.0 Hz, 2H), 3.65 (*t*, *J* = 6.0 Hz, 2H), 3.51 (*s*, 2H); ^13^C NMR (CD_3_OD, 100 MHz): δ 171.6, 166.5, 142.2, 139.7, 130.4, 129.5, 129.0, 128.3, 128.1, 126.7, 58.4, 51.4, 40.6.

## 6. Refinement

Crystal data, data collection and structure refinement details are summarized in Table 2 ▸. Most hydrogen atoms were fixed positionally in calculated positions using the AFIX command in *SHELXL* (Sheldrick 2015 ▸). These were refined as riding with distances of 0.95 Å for C—H, and 0.99 Å for CH_2_, and *U*_iso_ values for riding H atoms 1.2 times *U*_eq_(C) for both. OH atoms were also positionally fixed with *U*_iso_ values for riding H atoms 1.5 times *U*_eq_(C). The distances for O1—H1*a* and O1—H1*b* were restrained with a σ value of 0.02, and these were part of the positionally disordered water mol­ecule. The site occupancy factor was set with FVAR = 1 for H1*a* and H1*b*, and was set to a value of 0.5 for O5, O5*A*, H*a*, H*b*, H, and H*c*.

## Supplementary Material

Crystal structure: contains datablock(s) I. DOI: 10.1107/S2056989025010989/ej2016sup1.cif

Supporting information file. DOI: 10.1107/S2056989025010989/ej2016Isup3.cml

Supporting information file. DOI: 10.1107/S2056989025010989/ej2016sup2.txt

CCDC reference: 2513447

Additional supporting information:  crystallographic information; 3D view; checkCIF report

Additional supporting information:  crystallographic information; 3D view; checkCIF report

## Figures and Tables

**Table 1:** Hydrogen-bond geometry (Å, °)

*D*—H⋯*A*	*D*—H	H⋯*A*	*D*⋯*A*	*D*—H⋯*A*
O1—H1*a*⋯O3	0.84 (7)	2.1 (1)	2.742 (2)	131 (8)
O5*A*—H*b*⋯O3	0.87 (5)	2.03 (5)	2.897 (5)	176 (5)
O4—H4⋯O1	0.84 (1)	1.82 (1)	2.653 (3)	172 (2)
N2—H2⋯O5	0.85 (3)	2.05 (4)	2.855 (5)	158 (3)
O5—H⋯O5’	0.87 (3)	1.64 (3)	2.359 (6)	137 (5)
O5—H*a*⋯O2	0.87 (3)	1.88 (3)	2.744 (4)	176 (5)

**Table 2:** Experimental details

Crystal data	
Chemical formula	C_17_H_18_N_2_O_4_·H_2_O
*M*_r_	332.36
Crystal system, space group	Monoclinic, *P*2_1_/*n*
Temperature (K)	106
*a*, *b*, *c* (Å)	11.4354 (4), 6.9475 (2), 20.3670 (7)
β (°)	100.277 (1)
*V* (Å^3^)	1592.15 (9)
*Z*	4
Radiation type	Mo *K*α
μ (mm^−1^)	0.10
Crystal size (mm)	0.3 × 0.2 × 0.2

Data collection	
Diffractometer	Bruker APEXII CCD
Absorption correction	Multi-scan (*SADABS*; Krause *et al.*, 2015 ▸)
No. of measured, independent and observed [*I* ≥ 2u(*I*)] reflections	51035, 4905, 4763
*R*_int_	0.034
(sin θ/λ)_max_ (Å^−1^)	0.721

Refinement	
*R*[*F*^2^ > 2σ(*F*^2^)], *wR*(*F*^2^), *S*	0.077, 0.167, 0.97
No. of reflections	4905
No. of parameters	244
No. of restraints	2
H-atom treatment	H atoms treated by a mixture of independent and constrained refinement
Δρ_max_, Δρ_min_ (e Å^−3^)	0.48, −0.47

Computer programs: *APEX4* and *SAINT* V8.38A (Bruker, 2018 ▸), *OLEX2.solve* (Bourhis *et al.*, 2015 ▸); *OLEX2* (Dolomanov *et al.*, 2009 ▸) & *Mercury* (Macrae *et al.*, 2020 ▸).

## supplementary crystallographic information

### N-Hydroxy-4-{2-[(2-hydroxyethyl)(phenyl)amino]-2-oxoethyl}benzamide monohydrate Crystal data

**Table:**

C_17_H_18_N_2_O_4_·H_2_O	*F*(000) = 704.518
*M**_r_* = 332.36	*D*_x_ = 1.387 Mg m^−^^3^
Monoclinic, *P*2_1_/*n*	Mo *K*α radiation, λ = 0.71073 Å
*a* = 11.4354 (4) Å	Cell parameters from 9673 reflections
*b* = 6.9475 (2) Å	θ = 3.1–30.8°
*c* = 20.3670 (7) Å	µ = 0.10 mm^−^^1^
β = 100.277 (1)°	*T* = 106 K
*V* = 1592.15 (9) Å^3^	Prism, yellow
*Z* = 4	0.3 × 0.2 × 0.2 mm

### N-Hydroxy-4-{2-[(2-hydroxyethyl)(phenyl)amino]-2-oxoethyl}benzamide monohydrate Data collection

**Table:**

Bruker APEXII CCD diffractometer	4763 reflections with *I*≥ 2u(*I*)
φ and ω scans	*R*_int_ = 0.034
Absorption correction: multi-scan (SADABS; Krause et al., 2015)	θ_max_ = 30.8°, θ_min_ = 2.0°
*T*_min_ = 0.664, *T*_max_ = 0.746	*h* = −16→16
51035 measured reflections	*k* = −9→9
4905 independent reflections	*l* = −29→29

### N-Hydroxy-4-{2-[(2-hydroxyethyl)(phenyl)amino]-2-oxoethyl}benzamide monohydrate Refinement

**Table:**

Refinement on *F*^2^	2 restraints
Least-squares matrix: full	38 constraints
*R*[*F*^2^ > 2σ(*F*^2^)] = 0.077	H atoms treated by a mixture of independent and constrained refinement
*wR*(*F*^2^) = 0.167	*w* = 1/[σ^2^(*F*_o_^2^) + (0.*P*)^2^ + 7.424*P*] where *P* = (*F*_o_^2^ + 2*F*_c_^2^)/3
*S* = 0.97	(Δ/σ)_max_ = −0.001
4905 reflections	Δρ_max_ = 0.48 e Å^−^^3^
244 parameters	Δρ_min_ = −0.47 e Å^−^^3^

### N-Hydroxy-4-{2-[(2-hydroxyethyl)(phenyl)amino]-2-oxoethyl}benzamide monohydrate Fractional atomic coordinates and isotropic or equivalent isotropic displacement parameters (Å^2^)

**Table:**

	*x*	*y*	*z*	*U*_iso_*/*U*_eq_	Occ. (<1)
O1	0.00723 (16)	−0.2883 (3)	0.45116 (9)	0.0201 (4)	
H1a	0.043 (13)	−0.297 (8)	0.491 (3)	0.0302 (5)*	0.29 (11)
O3	0.77454 (15)	0.3314 (3)	0.46888 (9)	0.0233 (4)	
O4	0.84011 (16)	0.6689 (3)	0.52516 (9)	0.0239 (4)	
H4	0.8974 (5)	0.684 (6)	0.50495 (9)	0.0358 (6)*	
O2	0.26595 (15)	−0.0148 (3)	0.36080 (10)	0.0222 (4)	
N1	0.07493 (15)	0.0741 (3)	0.32907 (9)	0.0123 (3)	
N2	0.73517 (18)	0.6467 (3)	0.47879 (10)	0.0195 (4)	
O5	0.6027 (4)	0.9675 (7)	0.5137 (2)	0.0237 (9)	0.500000
H	0.5308 (17)	0.962 (11)	0.522 (3)	0.0355 (14)*	0.500000
Ha	0.647 (4)	0.982 (11)	0.5527 (12)	0.0355 (14)*	0.500000
C12	−0.01322 (17)	0.2117 (3)	0.29999 (10)	0.0116 (4)	
C17	−0.06824 (19)	0.3301 (3)	0.34063 (11)	0.0147 (4)	
H17	−0.04642 (19)	0.3229 (3)	0.38781 (11)	0.0177 (5)*	
C9	0.70516 (19)	0.4693 (3)	0.45758 (10)	0.0140 (4)	
C8	0.58347 (18)	0.4441 (3)	0.41677 (10)	0.0129 (4)	
C3	0.19332 (19)	0.1083 (3)	0.33641 (11)	0.0142 (4)	
C10	0.56656 (19)	0.2879 (3)	0.37312 (12)	0.0172 (4)	
H10	0.63229 (19)	0.2094 (3)	0.36791 (12)	0.0206 (5)*	
C13	−0.04569 (19)	0.2200 (3)	0.23093 (10)	0.0140 (4)	
H13	−0.00866 (19)	0.1377 (3)	0.20350 (10)	0.0167 (5)*	
C16	−0.1552 (2)	0.4586 (3)	0.31153 (12)	0.0169 (4)	
H16	−0.1927 (2)	0.5403 (3)	0.33891 (12)	0.0203 (5)*	
C6	0.37383 (19)	0.5159 (3)	0.38783 (11)	0.0145 (4)	
H6	0.30813 (19)	0.5950 (3)	0.39272 (11)	0.0174 (5)*	
C15	−0.18780 (19)	0.4683 (3)	0.24231 (11)	0.0161 (4)	
H15	−0.24764 (19)	0.5560 (3)	0.22262 (11)	0.0193 (5)*	
C7	0.48690 (19)	0.5615 (3)	0.42295 (10)	0.0141 (4)	
H7	0.49801 (19)	0.6719 (3)	0.45090 (10)	0.0169 (5)*	
C2	0.03527 (19)	−0.1112 (3)	0.35218 (11)	0.0148 (4)	
H2a	−0.04315 (19)	−0.1451 (3)	0.32556 (11)	0.0177 (5)*	
H2b	0.09244 (19)	−0.2132 (3)	0.34550 (11)	0.0177 (5)*	
C5	0.35599 (18)	0.3567 (3)	0.34581 (11)	0.0143 (4)	
C4	0.23250 (19)	0.3010 (3)	0.31170 (11)	0.0164 (4)	
H4a	0.23047 (19)	0.2933 (3)	0.26298 (11)	0.0197 (5)*	
H4b	0.17580 (19)	0.4021 (3)	0.31984 (11)	0.0197 (5)*	
C11	0.4544 (2)	0.2462 (4)	0.33716 (12)	0.0193 (5)	
H11	0.4445 (2)	0.1419 (4)	0.30650 (12)	0.0232 (5)*	
C14	−0.1328 (2)	0.3497 (3)	0.20221 (11)	0.0167 (4)	
H14	−0.1546 (2)	0.3571 (3)	0.15503 (11)	0.0200 (5)*	
C1	0.0259 (2)	−0.1006 (4)	0.42561 (12)	0.0210 (5)	
H1c	−0.0411 (2)	−0.0154 (4)	0.43120 (12)	0.0252 (6)*	
H1d	0.0998 (2)	−0.0447 (4)	0.45122 (12)	0.0252 (6)*	
O5A	0.6446 (4)	0.9970 (7)	0.5013 (2)	0.0224 (9)	0.500000
Hb	0.681 (5)	1.098 (6)	0.490 (3)	0.0336 (13)*	0.500000
Hc	0.5696 (12)	1.025 (9)	0.490 (4)	0.0336 (13)*	0.500000
H1b	0.076 (3)	−0.336 (7)	0.468 (3)	0.023 (15)*	0.71 (11)
H2	0.690 (3)	0.745 (5)	0.4777 (16)	0.025 (8)*	

### N-Hydroxy-4-{2-[(2-hydroxyethyl)(phenyl)amino]-2-oxoethyl}benzamide monohydrate Atomic displacement parameters (Å^2^)

**Table:**

	*U*^11^	*U*^22^	*U*^33^	*U*^12^	*U*^13^	*U*^23^
O1	0.0194 (8)	0.0182 (8)	0.0220 (8)	−0.0032 (7)	0.0015 (6)	0.0073 (7)
O3	0.0152 (8)	0.0278 (10)	0.0244 (9)	0.0079 (7)	−0.0033 (6)	−0.0055 (7)
O4	0.0175 (8)	0.0297 (10)	0.0210 (8)	−0.0096 (7)	−0.0057 (6)	0.0027 (7)
O2	0.0134 (7)	0.0167 (8)	0.0340 (10)	0.0034 (6)	−0.0022 (6)	0.0034 (7)
N1	0.0102 (7)	0.0105 (8)	0.0156 (8)	0.0002 (6)	0.0011 (6)	0.0020 (6)
N2	0.0148 (9)	0.0207 (10)	0.0203 (9)	−0.0044 (8)	−0.0041 (7)	0.0019 (8)
O5	0.017 (2)	0.024 (2)	0.028 (2)	0.0038 (18)	0.0001 (17)	−0.0013 (17)
C12	0.0086 (8)	0.0117 (9)	0.0141 (9)	0.0000 (7)	0.0009 (6)	−0.0002 (7)
C17	0.0155 (9)	0.0152 (10)	0.0138 (9)	0.0008 (8)	0.0030 (7)	−0.0011 (7)
C9	0.0126 (9)	0.0185 (10)	0.0111 (8)	−0.0011 (8)	0.0027 (7)	0.0013 (7)
C8	0.0113 (8)	0.0154 (9)	0.0122 (8)	−0.0014 (7)	0.0023 (7)	0.0021 (7)
C3	0.0117 (9)	0.0148 (9)	0.0154 (9)	0.0000 (7)	0.0006 (7)	−0.0009 (8)
C10	0.0115 (9)	0.0165 (10)	0.0239 (11)	−0.0001 (8)	0.0039 (8)	−0.0033 (8)
C13	0.0142 (9)	0.0145 (9)	0.0135 (9)	0.0005 (7)	0.0032 (7)	−0.0006 (7)
C16	0.0150 (9)	0.0161 (10)	0.0204 (10)	0.0036 (8)	0.0056 (8)	−0.0009 (8)
C6	0.0125 (9)	0.0153 (10)	0.0166 (9)	0.0015 (7)	0.0046 (7)	0.0030 (8)
C15	0.0120 (9)	0.0142 (9)	0.0212 (10)	0.0015 (8)	0.0011 (7)	0.0024 (8)
C7	0.0150 (9)	0.0142 (9)	0.0134 (9)	−0.0012 (7)	0.0036 (7)	0.0009 (7)
C2	0.0152 (9)	0.0109 (9)	0.0179 (9)	−0.0022 (7)	0.0023 (7)	0.0002 (7)
C5	0.0094 (8)	0.0165 (10)	0.0169 (9)	−0.0009 (7)	0.0017 (7)	0.0019 (8)
C4	0.0103 (8)	0.0176 (10)	0.0203 (10)	−0.0024 (8)	0.0001 (7)	0.0033 (8)
C11	0.0129 (9)	0.0202 (11)	0.0247 (11)	0.0002 (8)	0.0027 (8)	−0.0070 (9)
C14	0.0184 (10)	0.0163 (10)	0.0144 (9)	−0.0010 (8)	0.0002 (7)	0.0018 (8)
C1	0.0304 (12)	0.0152 (10)	0.0180 (10)	−0.0037 (9)	0.0058 (9)	0.0018 (8)
O5A	0.019 (2)	0.0181 (19)	0.028 (2)	−0.0002 (17)	−0.0015 (17)	0.0024 (16)

### N-Hydroxy-4-{2-[(2-hydroxyethyl)(phenyl)amino]-2-oxoethyl}benzamide monohydrate Geometric parameters (Å, º)

**Table:**

O1—H1a	0.8400	C13—H13	0.9500
O1—C1	1.434 (3)	C13—C14	1.392 (3)
O1—H1b	0.87 (3)	C16—H16	0.9500
O3—C9	1.239 (3)	C16—C15	1.394 (3)
O4—H4	0.8400	C6—H6	0.9500
O4—N2	1.397 (2)	C6—C7	1.398 (3)
O2—C3	1.234 (3)	C6—C5	1.391 (3)
N1—C12	1.438 (3)	C15—H15	0.9500
N1—C3	1.356 (3)	C15—C14	1.388 (3)
N1—C2	1.470 (3)	C7—H7	0.9500
N2—C9	1.330 (3)	C2—H2a	0.9900
N2—H2	0.85 (4)	C2—H2b	0.9900
O5—H	0.8689	C2—C1	1.520 (3)
O5—Ha	0.8701	C5—C4	1.509 (3)
C12—C17	1.394 (3)	C5—C11	1.399 (3)
C12—C13	1.390 (3)	C4—H4a	0.9900
C17—H17	0.9500	C4—H4b	0.9900
C17—C16	1.388 (3)	C11—H11	0.9500
C9—C8	1.498 (3)	C14—H14	0.9500
C8—C10	1.394 (3)	C1—H1c	0.9900
C8—C7	1.397 (3)	C1—H1d	0.9900
C3—C4	1.525 (3)	O5A—Hb	0.8705
C10—H10	0.9500	O5A—Hc	0.8701
C10—C11	1.389 (3)		
			
C1—O1—H1a	109.5	C5—C6—H6	119.48 (12)
H1b—O1—H1a	48.9	C5—C6—C7	121.0 (2)
H1b—O1—C1	108.0	H15—C15—C16	120.03 (13)
N2—O4—H4	109.5	C14—C15—C16	119.9 (2)
C3—N1—C12	122.78 (18)	C14—C15—H15	120.03 (13)
C2—N1—C12	118.71 (17)	C6—C7—C8	119.7 (2)
C2—N1—C3	118.50 (18)	H7—C7—C8	120.14 (12)
C9—N2—O4	117.5 (2)	H7—C7—C6	120.14 (13)
H2—N2—O4	112 (2)	H2a—C2—N1	109.52 (11)
H2—N2—C9	128 (2)	H2b—C2—N1	109.52 (11)
Ha—O5—H	104.5	H2b—C2—H2a	108.1
C17—C12—N1	120.33 (18)	C1—C2—N1	110.65 (18)
C13—C12—N1	119.05 (19)	C1—C2—H2a	109.52 (13)
C13—C12—C17	120.58 (19)	C1—C2—H2b	109.52 (13)
H17—C17—C12	120.31 (12)	C4—C5—C6	120.6 (2)
C16—C17—C12	119.4 (2)	C11—C5—C6	118.7 (2)
C16—C17—H17	120.31 (13)	C11—C5—C4	120.6 (2)
N2—C9—O3	122.4 (2)	C5—C4—C3	112.28 (18)
C8—C9—O3	120.9 (2)	H4a—C4—C3	109.15 (12)
C8—C9—N2	116.7 (2)	H4a—C4—C5	109.15 (12)
C10—C8—C9	117.02 (19)	H4b—C4—C3	109.15 (12)
C7—C8—C9	123.5 (2)	H4b—C4—C5	109.15 (13)
C7—C8—C10	119.35 (19)	H4b—C4—H4a	107.9
N1—C3—O2	120.7 (2)	C5—C11—C10	120.4 (2)
C4—C3—O2	121.69 (19)	H11—C11—C10	119.78 (14)
C4—C3—N1	117.63 (19)	H11—C11—C5	119.78 (13)
H10—C10—C8	119.72 (12)	C15—C14—C13	120.2 (2)
C11—C10—C8	120.6 (2)	H14—C14—C13	119.92 (13)
C11—C10—H10	119.72 (14)	H14—C14—C15	119.92 (13)
H13—C13—C12	120.20 (12)	C2—C1—O1	110.74 (19)
C14—C13—C12	119.6 (2)	H1c—C1—O1	109.50 (13)
C14—C13—H13	120.20 (13)	H1c—C1—C2	109.50 (13)
H16—C16—C17	119.84 (13)	H1d—C1—O1	109.50 (13)
C15—C16—C17	120.3 (2)	H1d—C1—C2	109.50 (13)
C15—C16—H16	119.84 (13)	H1d—C1—H1c	108.1
C7—C6—H6	119.48 (13)	Hc—O5A—Hb	104.4
			
O1—C1—C2—N1	−170.10 (18)	C17—C16—C15—C14	−0.3 (3)
O3—C9—N2—O4	−11.7 (3)	C9—C8—C10—C11	175.2 (2)
O3—C9—C8—C10	−22.8 (2)	C9—C8—C7—C6	−173.5 (2)
O3—C9—C8—C7	153.8 (2)	C8—C10—C11—C5	−2.0 (3)
O4—N2—C9—C8	170.35 (19)	C8—C7—C6—C5	−1.0 (2)
O2—C3—N1—C12	−179.0 (2)	C3—N1—C12—C13	83.9 (2)
O2—C3—N1—C2	1.3 (3)	C3—N1—C2—C1	87.2 (2)
O2—C3—C4—C5	−24.9 (2)	C3—C4—C5—C6	−113.8 (2)
N1—C12—C17—C16	−178.4 (2)	C3—C4—C5—C11	64.5 (2)
N1—C12—C13—C14	178.61 (19)	C10—C8—C7—C6	3.0 (2)
N1—C3—C4—C5	156.8 (2)	C10—C11—C5—C6	4.0 (3)
N2—C9—C8—C10	155.2 (2)	C10—C11—C5—C4	−174.4 (2)
N2—C9—C8—C7	−28.2 (2)	C13—C12—N1—C2	−96.4 (2)
C12—N1—C3—C4	−0.7 (2)	C13—C12—C17—C16	−0.7 (2)
C12—N1—C2—C1	−92.5 (2)	C13—C14—C15—C16	0.5 (3)
C12—C17—C16—C15	0.4 (3)	C7—C8—C10—C11	−1.5 (3)
C12—C13—C14—C15	−0.8 (3)	C7—C6—C5—C4	175.9 (2)
C17—C12—N1—C3	−98.3 (2)	C7—C6—C5—C11	−2.5 (3)
C17—C12—N1—C2	81.4 (2)	C2—N1—C3—C4	179.63 (19)
C17—C12—C13—C14	0.9 (2)		

### N-Hydroxy-4-{2-[(2-hydroxyethyl)(phenyl)amino]-2-oxoethyl}benzamide monohydrate Hydrogen-bond geometry (Å, º)

**Table:**

*D*—H···*A*	*D*—H	H···*A*	*D*···*A*	*D*—H···*A*
O1—H1*a*···O3	0.84 (7)	2.1 (1)	2.742 (2)	131 (8)
O5*A*—H*b*···O3	0.87 (5)	2.03 (5)	2.897 (5)	176 (5)
O4—H4···O1	0.84 (1)	1.82 (1)	2.653 (3)	172 (2)
N2—H2···O5	0.85 (3)	2.05 (4)	2.855 (5)	158 (3)
O5—H···O5′	0.87 (3)	1.64 (3)	2.359 (6)	137 (5)
O5—H*a*···O2	0.87 (3)	1.88 (3)	2.744 (4)	176 (5)
